# *tert*-Butyl *N*-{4-methyl-3-[4-(3-pyrid­yl)pyrimidin-2-yl­oxy]phen­yl}carbamate

**DOI:** 10.1107/S1600536809025604

**Published:** 2009-07-11

**Authors:** Shi-Gui Tang, Jian-Qiang Wang, Cheng Guo

**Affiliations:** a College of Science, Nanjing University of Technolgy, Xinmofan Road No. 5 Nanjing, Nanjing 210009, People’s Republic of China

## Abstract

In the mol­ecule of the title compound, C_21_H_22_N_4_O_3_, the pyrimidine ring is oriented at dihedral angles of 0.51 (3) and 50.76 (3)° to the pyridine and benzene rings, respectively. In the crystal structure, inter­molecular N—H⋯N hydrogen bonds link the mol­ecules into centrosymmetric dimers, forming *R*_2_^2^(24) ring motifs; the dimers are linked by inter­molecular C—H⋯O hydrogen bonds into a two-dimensional network. π–π contacts between the benzene rings and between the pyrimidine and pyridine rings [centroid–centroid distances = 3.891 (1) and 3.646 (1) Å, respectively] may further stabilize the structure. Two weak C—H⋯π inter­actions are also present.

## Related literature

For bond-length data, see: Allen *et al.* (1987 ▶). For ring-motifs, see: Bernstein *et al.* (1995 ▶).
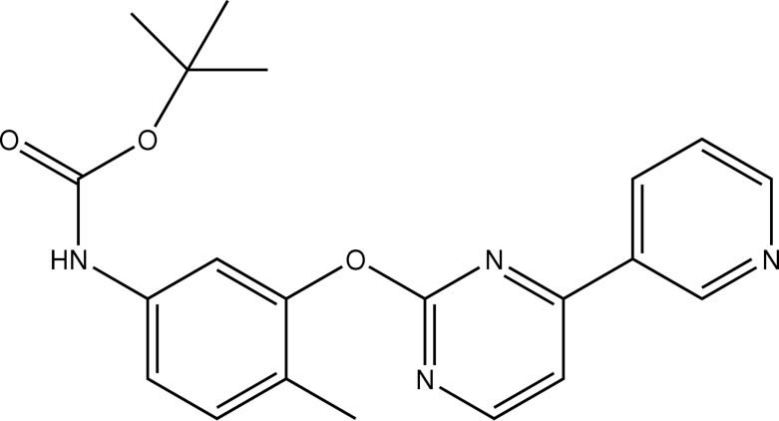


## Experimental

### 

#### Crystal data

C_21_H_22_N_4_O_3_
*M* *_r_* = 378.43
Triclinic, 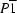

*a* = 9.951 (2) Å
*b* = 10.733 (2) Å
*c* = 11.577 (2) Å
α = 114.74 (3)°
β = 107.14 (3)°
γ = 99.97 (3)°
*V* = 1008.6 (6) Å^3^
*Z* = 2
Mo *K*α radiation
μ = 0.09 mm^−1^
*T* = 294 K
0.30 × 0.20 × 0.10 mm

#### Data collection

Enraf–Nonius CAD-4 diffractometer
Absorption correction: ψ scan (North *et al.*, 1968 ▶) *T*_min_ = 0.975, *T*_max_ = 0.992
3882 measured reflections
3652 independent reflections
2333 reflections with *I* > 2σ(*I*)
*R*_int_ = 0.026
3 standard reflections frequency: 120 min intensity decay: 1%

#### Refinement

*R*[*F* ^2^ > 2σ(*F* ^2^)] = 0.058
*wR*(*F* ^2^) = 0.184
*S* = 1.01
3652 reflections
254 parameters
H-atom parameters constrained
Δρ_max_ = 0.29 e Å^−3^
Δρ_min_ = −0.27 e Å^−3^

### 

Data collection: *CAD-4 Software* (Enraf–Nonius, 1989 ▶); cell refinement: *CAD-4 Software*; data reduction: *XCAD4* (Harms & Wocadlo, 1995 ▶); program(s) used to solve structure: *SHELXS97* (Sheldrick, 2008 ▶); program(s) used to refine structure: *SHELXL97* (Sheldrick, 2008 ▶); molecular graphics: *ORTEP-3 for Windows* (Farrugia, 1997 ▶); software used to prepare material for publication: *SHELXL97* and *PLATON* (Spek, 2009 ▶).

## Supplementary Material

Crystal structure: contains datablocks global, I. DOI: 10.1107/S1600536809025604/hk2725sup1.cif

Structure factors: contains datablocks I. DOI: 10.1107/S1600536809025604/hk2725Isup2.hkl

Additional supplementary materials:  crystallographic information; 3D view; checkCIF report

## Figures and Tables

**Table 1:** Hydrogen-bond geometry (Å, °)

*D*—H⋯*A*	*D*—H	H⋯*A*	*D*⋯*A*	*D*—H⋯*A*
N1—H1*A*⋯N4^i^	0.86	2.10	2.944 (4)	165
C15—H15*A*⋯O2^ii^	0.93	2.45	3.382 (4)	177
C18—H18*A*⋯O2^ii^	0.93	2.39	3.319 (4)	174
C3—H3*B*⋯*Cg*1^i^	0.96	2.86	3.560 (3)	131
C12—H12*B*⋯*Cg*2^iii^	0.96	2.90	3.788 (3)	154

Symmetry codes: (i) 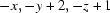; (ii) 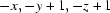; (iii) 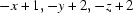. *Cg*1 and *Cg*2 are the centroids of the C6–C11 and N2/N3/C13–C16 rings, respectively.

**Fig. 1.**
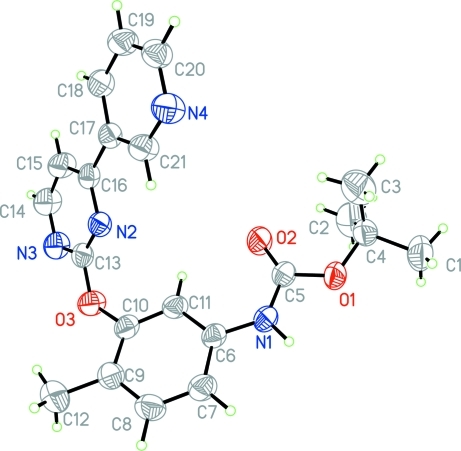
The molecular structure of the title molecule, with the atom-numbering scheme. Displacement ellipsoids are drawn at the 30% probability level.

**Fig. 2.**
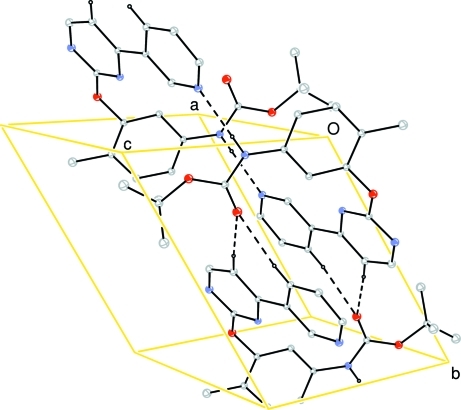
A partial packing diagram of the title compound. Hydrogen bonds are shown as dashed lines.

## supplementary crystallographic information

### Comment

Some derivatives of phenol are important chemical materials. We report herein the crystal structure of the title compound.

In the molecule of the title compound (Fig 1), the bond lengths (Allen *et al.*, 1987) and angles are within normal ranges. Rings A (C6-C11), B (N2/N3/C13-C16) and C (N4/C17-C21) are, of course, planar and the dihedral angles between them are A/B = 50.76 (3), A/C = 50.58 (3) and B/C = 0.51 (3) °.

In the crystal structure, intermolecular N-H···N hydrogen bonds (Table 1) link the molecules into centrosymmetric dimers forming *R*_2_^2^(24) ring motifs (Bernstein *et al.*, 1995), and then intermolecular C-H···O hydrogen bonds (Table 1) link them into a two dimensional network (Fig. 2), in which they may be effective in the stabilization of the structure. The π–π contacts between the phenyl rings and between the pyrimidine and the pyridine rings, Cg1—Cg1^i^ and Cg2—Cg3^ii^ [symmetry codes: (i) 1 - x, 2 - y, 1 - z, (ii) -x, 1 - y, 1 - z, where Cg1, Cg2 and Cg3 are centroids of the rings A (C6-C11), B (N2/N3/C13-C16) and C (N4/C17-C21), respectively] may further stabilize the structure, with centroid-centroid distances of 3.891 (1) and 3.646 (1) Å, respectively. There also exist two weak C—H···π interactions (Table 1).

### Experimental

To a mixture of 2-(methylsulfonyl)-4-(pyridin-3-yl)pyrimidine (47.1 g, 0.2 mol) in DMF (75 ml) and *tert*-butyl-3-hydroxy-4-methylphenylcarbamate (44.7 g, 0.2 mol) in DMF (150 ml) was added sodium hydride (44.4 g) slowly and was stirred for 18 h at room temperature. After acidified with citric acid the reaction mixture was poured into ice-water (2000 ml). The precipitate was filtered, washed with water and was extracted with dichloromethane.The dichloromethane layer was dried over anhydrous magnesium sulfate and evaporated *in vacuo*. The residue was recrystallized from ethyl ether to give the title compound (yield; 73.4 g). Crystals suitable for X-ray analysis were obtained by slow evaporation of an ethanol solution.

### Refinement

H atoms were positioned geometrically with N-H = 0.86 Å (for NH) and C-H = 0.93 and 0.96 Å for aromatic and methyl H atoms, respectively, and constrained to ride on their parent atoms, with U_iso_(H) = xU_eq_(C,N), where x = 1.5 for methyl H and x = 1.2 for all other H atoms.

### Crystal data

**Table:**

C_21_H_22_N_4_O_3_	*Z* = 2
*M**_r_* = 378.43	*F*(000) = 400
Triclinic, *P*1	*D*_x_ = 1.246 Mg m^−^^3^
Hall symbol: -P 1	Mo *K*α radiation, λ = 0.71073 Å
*a* = 9.951 (2) Å	Cell parameters from 25 reflections
*b* = 10.733 (2) Å	θ = 9–13°
*c* = 11.577 (2) Å	µ = 0.09 mm^−^^1^
α = 114.74 (3)°	*T* = 294 K
β = 107.14 (3)°	Block, colorless
γ = 99.97 (3)°	0.30 × 0.20 × 0.10 mm
*V* = 1008.6 (6) Å^3^	

### Data collection

**Table:**

Enraf–Nonius CAD-4 diffractometer	2333 reflections with *I* > 2σ(*I*)
Radiation source: fine-focus sealed tube	*R*_int_ = 0.026
graphite	θ_max_ = 25.3°, θ_min_ = 2.1°
ω/2θ scans	*h* = 0→11
Absorption correction: ψ scan (North *et al.*, 1968)	*k* = −12→12
*T*_min_ = 0.975, *T*_max_ = 0.992	*l* = −13→13
3882 measured reflections	3 standard reflections every 120 min
3652 independent reflections	intensity decay: 1%

### Refinement

**Table:**

Refinement on *F*^2^	Secondary atom site location: difference Fourier map
Least-squares matrix: full	Hydrogen site location: inferred from neighbouring sites
*R*[*F*^2^ > 2σ(*F*^2^)] = 0.058	H-atom parameters constrained
*wR*(*F*^2^) = 0.184	*w* = 1/[σ^2^(*F*_o_^2^) + (0.1*P*)^2^ + 0.07*P*] where *P* = (*F*_o_^2^ + 2*F*_c_^2^)/3
*S* = 1.00	(Δ/σ)_max_ < 0.001
3652 reflections	Δρ_max_ = 0.29 e Å^−^^3^
254 parameters	Δρ_min_ = −0.27 e Å^−^^3^
0 restraints	Extinction correction: *SHELXL97* (Sheldrick, 2008), Fc^*^=kFc[1+0.001xFc^2^λ^3^/sin(2θ)]^-1/4^
Primary atom site location: structure-invariant direct methods	Extinction coefficient: 0.033 (5)

### Special details

**Table:**

Geometry. All e.s.d.'s (except the e.s.d. in the dihedral angle between two l.s. planes) are estimated using the full covariance matrix. The cell e.s.d.'s are taken into account individually in the estimation of e.s.d.'s in distances, angles and torsion angles; correlations between e.s.d.'s in cell parameters are only used when they are defined by crystal symmetry. An approximate (isotropic) treatment of cell e.s.d.'s is used for estimating e.s.d.'s involving l.s. planes.
Refinement. Refinement of *F*^2^ against ALL reflections. The weighted *R*-factor *wR* and goodness of fit *S* are based on *F*^2^, conventional *R*-factors *R* are based on *F*, with *F* set to zero for negative *F*^2^. The threshold expression of *F*^2^ > σ(*F*^2^) is used only for calculating *R*-factors(gt) *etc*. and is not relevant to the choice of reflections for refinement. *R*-factors based on *F*^2^ are statistically about twice as large as those based on *F*, and *R*- factors based on ALL data will be even larger.

### Fractional atomic coordinates and isotropic or equivalent isotropic displacement parameters (Å^2^)

**Table:**

	*x*	*y*	*z*	*U*_iso_*/*U*_eq_	
O1	0.0153 (2)	0.8866 (2)	0.2102 (2)	0.0600 (6)	
O2	0.0613 (2)	0.7528 (2)	0.3167 (2)	0.0719 (7)	
O3	0.4840 (2)	0.8449 (2)	0.7311 (2)	0.0594 (6)	
N1	0.2087 (2)	0.9880 (2)	0.4085 (2)	0.0498 (6)	
H1A	0.2141	1.0599	0.3929	0.060*	
N2	0.2426 (2)	0.7463 (2)	0.7031 (2)	0.0429 (5)	
N3	0.4351 (3)	0.6437 (3)	0.7463 (3)	0.0574 (7)	
N4	−0.1874 (3)	0.7568 (2)	0.6228 (3)	0.0567 (7)	
C1	−0.1644 (4)	0.8538 (4)	0.0096 (4)	0.0910 (12)	
H1B	−0.1731	0.9458	0.0640	0.136*	
H1C	−0.2570	0.7920	−0.0708	0.136*	
H1D	−0.0855	0.8694	−0.0200	0.136*	
C2	−0.1138 (4)	0.6358 (4)	0.0150 (4)	0.0862 (11)	
H2B	−0.0922	0.5919	0.0724	0.129*	
H2C	−0.0339	0.6503	−0.0136	0.129*	
H2D	−0.2057	0.5727	−0.0659	0.129*	
C3	−0.2421 (4)	0.7685 (5)	0.1575 (4)	0.0929 (12)	
H3B	−0.2485	0.8630	0.2080	0.139*	
H3C	−0.2119	0.7312	0.2196	0.139*	
H3D	−0.3381	0.7032	0.0834	0.139*	
C4	−0.1288 (3)	0.7813 (3)	0.0971 (3)	0.0550 (8)	
C5	0.0919 (3)	0.8649 (3)	0.3128 (3)	0.0490 (7)	
C6	0.3229 (3)	1.0147 (3)	0.5304 (3)	0.0417 (6)	
C7	0.4275 (3)	1.1551 (3)	0.6155 (3)	0.0515 (7)	
H7A	0.4202	1.2258	0.5898	0.062*	
C8	0.5415 (3)	1.1899 (3)	0.7372 (3)	0.0573 (8)	
H8A	0.6102	1.2844	0.7926	0.069*	
C9	0.5577 (3)	1.0880 (3)	0.7807 (3)	0.0505 (7)	
C10	0.4535 (3)	0.9502 (3)	0.6937 (3)	0.0449 (6)	
C11	0.3367 (3)	0.9096 (3)	0.5703 (3)	0.0459 (7)	
H11A	0.2689	0.8147	0.5150	0.055*	
C12	0.6828 (4)	1.1258 (4)	0.9146 (3)	0.0766 (10)	
H12A	0.6742	1.0413	0.9256	0.115*	
H12B	0.6764	1.2028	0.9920	0.115*	
H12C	0.7773	1.1573	0.9114	0.115*	
C13	0.3789 (3)	0.7420 (3)	0.7256 (3)	0.0446 (6)	
C14	0.3369 (3)	0.5396 (3)	0.7410 (3)	0.0610 (8)	
H14A	0.3692	0.4692	0.7562	0.073*	
C15	0.1907 (3)	0.5281 (3)	0.7147 (3)	0.0569 (8)	
H15A	0.1244	0.4510	0.7090	0.068*	
C16	0.1453 (3)	0.6373 (3)	0.6967 (2)	0.0404 (6)	
C17	−0.0094 (3)	0.6389 (3)	0.6684 (3)	0.0404 (6)	
C18	−0.1188 (3)	0.5330 (3)	0.6602 (3)	0.0507 (7)	
H18A	−0.0970	0.4574	0.6725	0.061*	
C19	−0.2597 (3)	0.5405 (3)	0.6339 (3)	0.0571 (8)	
H19A	−0.3347	0.4697	0.6277	0.068*	
C20	−0.2896 (3)	0.6538 (3)	0.6167 (3)	0.0525 (7)	
H20A	−0.3854	0.6584	0.6000	0.063*	
C21	−0.0505 (3)	0.7479 (3)	0.6477 (3)	0.0519 (7)	
H21A	0.0217	0.8189	0.6514	0.062*	

### Atomic displacement parameters (Å^2^)

**Table:**

	*U*^11^	*U*^22^	*U*^33^	*U*^12^	*U*^13^	*U*^23^
O1	0.0613 (12)	0.0482 (11)	0.0602 (12)	0.0112 (10)	0.0039 (10)	0.0353 (10)
O2	0.0703 (14)	0.0469 (12)	0.0834 (15)	0.0087 (10)	0.0013 (12)	0.0443 (11)
O3	0.0419 (11)	0.0666 (13)	0.0880 (15)	0.0232 (10)	0.0201 (10)	0.0567 (12)
N1	0.0556 (14)	0.0365 (12)	0.0589 (14)	0.0166 (11)	0.0147 (12)	0.0307 (11)
N2	0.0440 (13)	0.0406 (12)	0.0466 (13)	0.0166 (10)	0.0146 (10)	0.0258 (10)
N3	0.0573 (15)	0.0581 (15)	0.0733 (17)	0.0319 (13)	0.0248 (13)	0.0435 (14)
N4	0.0489 (14)	0.0506 (14)	0.0806 (18)	0.0231 (12)	0.0241 (13)	0.0406 (14)
C1	0.089 (3)	0.086 (3)	0.079 (2)	0.018 (2)	0.000 (2)	0.053 (2)
C2	0.101 (3)	0.064 (2)	0.071 (2)	0.030 (2)	0.023 (2)	0.0216 (19)
C3	0.068 (2)	0.119 (3)	0.106 (3)	0.039 (2)	0.037 (2)	0.066 (3)
C4	0.0508 (17)	0.0516 (17)	0.0580 (18)	0.0161 (14)	0.0135 (15)	0.0299 (15)
C5	0.0509 (16)	0.0428 (15)	0.0578 (17)	0.0181 (13)	0.0175 (14)	0.0313 (14)
C6	0.0423 (14)	0.0407 (14)	0.0512 (16)	0.0192 (12)	0.0210 (13)	0.0275 (13)
C7	0.0546 (17)	0.0417 (15)	0.0616 (18)	0.0178 (13)	0.0215 (15)	0.0296 (14)
C8	0.0544 (17)	0.0427 (16)	0.0601 (18)	0.0088 (13)	0.0149 (15)	0.0219 (14)
C9	0.0457 (15)	0.0533 (17)	0.0540 (17)	0.0176 (14)	0.0182 (13)	0.0293 (14)
C10	0.0398 (14)	0.0521 (16)	0.0591 (17)	0.0218 (13)	0.0230 (13)	0.0375 (14)
C11	0.0455 (15)	0.0396 (14)	0.0556 (17)	0.0150 (12)	0.0194 (13)	0.0271 (13)
C12	0.066 (2)	0.079 (2)	0.065 (2)	0.0115 (18)	0.0066 (17)	0.0370 (19)
C13	0.0442 (15)	0.0469 (15)	0.0458 (15)	0.0180 (12)	0.0138 (12)	0.0278 (13)
C14	0.064 (2)	0.0559 (18)	0.086 (2)	0.0355 (16)	0.0306 (17)	0.0495 (18)
C15	0.0608 (19)	0.0468 (16)	0.078 (2)	0.0229 (14)	0.0281 (16)	0.0424 (16)
C16	0.0502 (15)	0.0341 (13)	0.0380 (14)	0.0169 (12)	0.0157 (12)	0.0193 (11)
C17	0.0445 (15)	0.0343 (13)	0.0423 (14)	0.0150 (11)	0.0142 (12)	0.0210 (11)
C18	0.0527 (17)	0.0448 (15)	0.0610 (18)	0.0179 (13)	0.0187 (14)	0.0343 (14)
C19	0.0512 (17)	0.0516 (17)	0.072 (2)	0.0109 (14)	0.0213 (15)	0.0392 (16)
C20	0.0439 (15)	0.0542 (17)	0.0623 (18)	0.0186 (13)	0.0200 (14)	0.0321 (15)
C21	0.0466 (16)	0.0412 (15)	0.073 (2)	0.0157 (12)	0.0196 (14)	0.0363 (15)

### Geometric parameters (Å, °)

**Table:**

O1—C4	1.463 (3)	C6—C7	1.391 (4)
O1—C5	1.344 (3)	C6—C11	1.399 (3)
O2—C5	1.211 (3)	C7—C8	1.373 (4)
O3—C10	1.418 (3)	C7—H7A	0.9300
O3—C13	1.349 (3)	C8—C9	1.396 (4)
N1—C5	1.345 (3)	C8—H8A	0.9300
N1—C6	1.403 (3)	C9—C10	1.372 (4)
N1—H1A	0.8600	C9—C12	1.509 (4)
N3—C13	1.349 (3)	C10—C11	1.380 (4)
N3—C14	1.318 (4)	C11—H11A	0.9300
N4—C20	1.327 (3)	C12—H12A	0.9600
N4—C21	1.336 (3)	C12—H12B	0.9600
C1—C4	1.518 (4)	C12—H12C	0.9600
C1—H1B	0.9600	C14—C15	1.367 (4)
C1—H1C	0.9600	C14—H14A	0.9300
C1—H1D	0.9600	C15—C16	1.397 (3)
N2—C13	1.317 (3)	C15—H15A	0.9300
N2—C16	1.344 (3)	C16—C17	1.482 (4)
C2—C4	1.512 (4)	C17—C18	1.380 (4)
C2—H2B	0.9600	C17—C21	1.391 (3)
C2—H2C	0.9600	C18—C19	1.368 (4)
C2—H2D	0.9600	C18—H18A	0.9300
C3—C4	1.507 (5)	C19—C20	1.379 (4)
C3—H3B	0.9600	C19—H19A	0.9300
C3—H3C	0.9600	C20—H20A	0.9300
C3—H3D	0.9600	C21—H21A	0.9300
			
C5—O1—C4	122.7 (2)	C9—C8—H8A	118.9
C13—O3—C10	123.90 (19)	C10—C9—C8	116.0 (3)
C5—N1—C6	128.6 (2)	C10—C9—C12	121.7 (3)
C5—N1—H1A	115.7	C8—C9—C12	122.3 (3)
C6—N1—H1A	115.7	C9—C10—C11	124.2 (2)
C13—N2—C16	116.2 (2)	C9—C10—O3	114.6 (2)
C14—N3—C13	113.6 (2)	C11—C10—O3	120.9 (2)
C4—C1—H1B	109.5	C10—C11—C6	118.4 (2)
C4—C1—H1C	109.5	C10—C11—H11A	120.8
H1B—C1—H1C	109.5	C6—C11—H11A	120.8
C4—C1—H1D	109.5	C9—C12—H12A	109.5
H1B—C1—H1D	109.5	C9—C12—H12B	109.5
H1C—C1—H1D	109.5	H12A—C12—H12B	109.5
C4—C2—H2B	109.5	C9—C12—H12C	109.5
C4—C2—H2C	109.5	H12A—C12—H12C	109.5
H2B—C2—H2C	109.5	H12B—C12—H12C	109.5
C4—C2—H2D	109.5	N2—C13—O3	120.7 (2)
H2B—C2—H2D	109.5	N2—C13—N3	128.4 (3)
H2C—C2—H2D	109.5	O3—C13—N3	110.9 (2)
C4—C3—H3B	109.5	N3—C14—C15	124.3 (3)
C4—C3—H3C	109.5	N3—C14—H14A	117.8
H3B—C3—H3C	109.5	C15—C14—H14A	117.8
C4—C3—H3D	109.5	C14—C15—C16	117.1 (3)
H3B—C3—H3D	109.5	C14—C15—H15A	121.4
H3C—C3—H3D	109.5	C16—C15—H15A	121.4
C20—N4—C21	117.2 (2)	N2—C16—C15	120.4 (2)
O1—C4—C3	109.0 (3)	N2—C16—C17	116.8 (2)
O1—C4—C2	110.8 (2)	C15—C16—C17	122.8 (2)
C3—C4—C2	112.3 (3)	C18—C17—C21	117.2 (2)
O1—C4—C1	102.0 (2)	C18—C17—C16	122.0 (2)
C3—C4—C1	111.1 (3)	C21—C17—C16	120.8 (2)
C2—C4—C1	111.1 (3)	C19—C18—C17	119.3 (2)
O2—C5—O1	125.6 (3)	C19—C18—H18A	120.3
O2—C5—N1	126.3 (3)	C17—C18—H18A	120.3
O1—C5—N1	108.1 (2)	C18—C19—C20	119.4 (3)
C7—C6—C11	118.9 (2)	C18—C19—H19A	120.3
C7—C6—N1	117.0 (2)	C20—C19—H19A	120.3
C11—C6—N1	124.0 (2)	N4—C20—C19	122.8 (3)
C8—C7—C6	120.3 (3)	N4—C20—H20A	118.6
C8—C7—H7A	119.8	C19—C20—H20A	118.6
C6—C7—H7A	119.8	N4—C21—C17	124.0 (2)
C7—C8—C9	122.2 (3)	N4—C21—H21A	118.0
C7—C8—H8A	118.9	C17—C21—H21A	118.0
			
C5—O1—C4—C3	64.0 (3)	C16—N2—C13—O3	178.7 (2)
C5—O1—C4—C2	−60.1 (4)	C16—N2—C13—N3	−1.6 (4)
C5—O1—C4—C1	−178.5 (3)	C10—O3—C13—N2	−9.7 (4)
C4—O1—C5—O2	8.5 (5)	C10—O3—C13—N3	170.6 (2)
C4—O1—C5—N1	−172.4 (2)	C14—N3—C13—N2	0.8 (4)
C6—N1—C5—O2	2.1 (5)	C14—N3—C13—O3	−179.5 (2)
C6—N1—C5—O1	−177.0 (2)	C13—N3—C14—C15	1.1 (5)
C5—N1—C6—C7	−177.0 (3)	N3—C14—C15—C16	−2.0 (5)
C5—N1—C6—C11	2.9 (4)	C13—N2—C16—C15	0.5 (4)
C11—C6—C7—C8	−0.6 (4)	C13—N2—C16—C17	−178.7 (2)
N1—C6—C7—C8	179.3 (3)	C14—C15—C16—N2	1.1 (4)
C6—C7—C8—C9	0.0 (5)	C14—C15—C16—C17	−179.7 (3)
C7—C8—C9—C10	0.6 (4)	N2—C16—C17—C18	−179.7 (2)
C7—C8—C9—C12	−179.9 (3)	C15—C16—C17—C18	1.1 (4)
C8—C9—C10—C11	−0.7 (4)	N2—C16—C17—C21	0.9 (4)
C12—C9—C10—C11	179.9 (3)	C15—C16—C17—C21	−178.3 (3)
C8—C9—C10—O3	172.1 (2)	C21—C17—C18—C19	−0.6 (4)
C12—C9—C10—O3	−7.3 (4)	C16—C17—C18—C19	180.0 (2)
C13—O3—C10—C9	138.8 (3)	C17—C18—C19—C20	−0.3 (4)
C13—O3—C10—C11	−48.2 (4)	C21—N4—C20—C19	−0.4 (4)
C9—C10—C11—C6	0.1 (4)	C18—C19—C20—N4	0.8 (5)
O3—C10—C11—C6	−172.3 (2)	C20—N4—C21—C17	−0.6 (4)
C7—C6—C11—C10	0.6 (4)	C18—C17—C21—N4	1.1 (4)
N1—C6—C11—C10	−179.3 (2)	C16—C17—C21—N4	−179.5 (3)

### Hydrogen-bond geometry (Å, °)

**Table:**

*D*—H···*A*	*D*—H	H···*A*	*D*···*A*	*D*—H···*A*
N1—H1A···N4^i^	0.86	2.10	2.944 (4)	165
C15—H15A···O2^ii^	0.93	2.45	3.382 (4)	177
C18—H18A···O2^ii^	0.93	2.39	3.319 (4)	174
C3—H3B···Cg1^i^	0.96	2.86	3.560 (3)	131
C12—H12B···Cg2^iii^	0.96	2.90	3.788 (3)	154

Symmetry codes: (i) −*x*, −*y*+2, −*z*+1; (ii) −*x*, −*y*+1, −*z*+1; (iii) −*x*+1, −*y*+2, −*z*+2.
